# Splicing factor derived circular RNA *circCAMSAP1* accelerates nasopharyngeal carcinoma tumorigenesis via a SERPINH1/c-Myc positive feedback loop

**DOI:** 10.1186/s12943-022-01502-2

**Published:** 2022-02-28

**Authors:** Yian Wang, Qijia Yan, Yongzhen Mo, Yuhang Liu, Yumin Wang, Shanshan Zhang, Can Guo, Fuyan Wang, Guiyuan Li, Zhaoyang Zeng, Wei Xiong

**Affiliations:** 1 NHC Key Laboratory of Carcinogenesis, Hunan Cancer Hospital and the Affiliated Cancer Hospital of Xiangya School of Medicine, Central South University, Changsha, China; 2 Key Laboratory of Carcinogenesis and Cancer Invasion of the Chinese Ministry of Education, Cancer Research Institute, Central South University, Changsha, China; 3 Department of Otolaryngology Head and Neck Surgery, Xiangya Hospital, Central South University, Changsha, China; 4 Department of stomatology, Xiangya Hospital, Central South University, Changsha, China; 5 Hunan Key Laboratory of Nonresolving Inflammation and Cancer, Disease Genome Research Center, The Third Xiangya Hospital, Central South University, Changsha, China

**Keywords:** Nasopharyngeal carcinoma, *circCAMSAP1*, SERPINH1, c-Myc, SRSF10, Proliferation, Metastasis

## Abstract

**Background:**

Circular RNAs play an important role in tumor genesis and progression, but they have not been sufficiently studied in patients with nasopharyngeal carcinoma (NPC).

**Methods:**

The circular RNA, *circCAMSAP1,* was screened in NPC cells by RNA sequencing analysis. The expression of *circCAMSAP1* in NPC tissues was examined by real-time quantitative polymerase chain reaction (RT-qPCR) and in situ hybridization. Wound-healing, transwell, MTT and flow cytometry assays, and nude mouse tumor models were used to explore the effect of *circCAMSAP1* on proliferation and metastasis of NPC in vitro or in vivo. The downstream proteins regulated by *circCAMSAP1* were screened using mass spectrometry. The interaction between *circCAMSAP1* and the *SERPINH1* mRNA was identified using the circular RNA immunoprecipitation method and the luciferase reporter assay. The interaction between SERPINH1 and transcription factor c-Myc was verified through Co-immunoprecipitation (Co-IP) and immunofluorescence. The effect of c-Myc on the generation of *circCAMSAP1* was examined through RT-qPCR and chromatin immunoprecipitation. Finally, the splicing factors that promote the production of *circCAMSAP1* were explored by RT-qPCR and RNA immunoprecipitation (RIP).

**Results:**

We found that *circCAMSAP1* was highly expressed in NPC tissues and promoted NPC proliferation and metastasis. Additionally, *circCAMSAP1* promoted SERPINH1 expression through improved *SERPINH1* mRNA stability by binding to the 3′-untranslated region (3’UTR) of *SERPINH1*. Highly expressed SERPINH1 reduced the ubiquitination-degradation rate of c-Myc, causing increased tumorigenesis. Meanwhile, c-Myc, cooperating with splicing factor 10 (SRSF10), could also promote *CAMSAP1* pre-mRNA transcription and back-splicing, forming a positive feedback of *circCAMSAP1* production, resulting in the proliferation and metastasis of NPC.

**Conclusions:**

Our findings revealed that *circCAMSAP1* promotes NPC proliferation and metastasis by binding to the 3’UTR of *SERPINH1*, suggesting that the positive feedback of *circCAMSAP1*-SERPINH1-c-Myc may serve as a prognostic biomarker or therapeutic target in patients with NPC.

**Supplementary Information:**

The online version contains supplementary material available at 10.1186/s12943-022-01502-2.

## Background

Nasopharyngeal carcinoma (NPC) is a common head and neck malignant tumor in southern China [1]. Factors contributing to the pathogenesis of NPC mainly include Epstein-Barr virus infection [2], genetic factors [3], and environmental factors [4]. The most common pathological type of NPC is non-keratinizing carcinoma with a high degree of malignancy, metastasis, and proliferative ability [5]. The onset of NPC is hard to detect, so early diagnosis is rare, and NPC has often already progressed into middle or late stage by the time it is detected [6]. While radiotherapy and chemotherapy can effectively control the growth of local tumors, local recurrence and distant metastasis contribute to the low survival rate of patients with NPC [7]. Therefore, finding biomarkers for early diagnosis of NPC and exploring the molecular mechanisms of metastasis and proliferation are essential for optimizing clinical treatment strategies.

Circular RNAs, or circRNAs, are non-coding RNAs that have recently received widespread attention. They were originally regarded as a by-product of mRNA production from the processing of precursor mRNA [8]. With the help of splicing factors, the 3′ end and 5′ end are reversely spliced to form a closed loop; this unique structure of circRNAs prevents their degradation by RNase [9]. Additionally, circRNAs are regarded as potential markers of cancer [10]. Increasingly more circRNAs are found to be involved in the growth and metastasis of a variety of malignant tumors, and are closely related to the recovery of the disease [11]. CircRNAs can function by interacting with RNA [12, 13], DNA [14], or proteins [15], or encode small peptides [16] to participate in intracellular signal transduction. Our previous studies have found that several circRNAs have played an important role in the metastasis and proliferation of NPC [17–20], but further studies searching for more such circRNAs are warranted.

In this study, we found that the circRNA *circCAMSAP1* is highly expressed in patients with NPC tissues, and strongly associated with the clinical and TNM stages of NPC patients. Furthermore, we found that *circCAMSAP1* affects the proliferation and metastasis of NPC in vitro and in vivo. We also revealed that *circCAMSAP1* promotes SERPINH1 expression and improves *SERPINH1* mRNA stability by binding to its 3’-untranslated region (3’UTR). Additionally, c-Myc was found to be involved in the transcription and formation of *circCAMSAP1*, cooperating with SRSF10. Therefore, *circCAMSAP1*-SERPINH1-c-Myc forms a positive feedback loop and may be a novel therapeutic target for treatment of NPC.

## Methods

### Data analysis

The RNA sequencing data of clinical NPC samples (GSE68799) was downloaded from the Gene Expression Omnibus (GEO) database. The reads per million (RPM) value was used to standardize the count value for subsequent analysis. The NPC gene expression data (GSE12452, GSE53819) were used to analyze the expression of SERPINH1 and SRSF10.

### Tissue samples

NPC tissue samples from two cohorts were collected at the Xiangya Hospital of Central South University. The first cohort included 29 NPC tissues and 12 chronic nasopharyngeal inflammation tissues (Table S1) for RT-qPCR. The second cohort included 82 NPC tissues and adjacent non-NPC tissues (Table S2) for in situ hybridization. The study was approved by the Joint Ethics Committee of the Health Bureau of Central South University, and the written informed consent was provided by each participant prior to surgery.

### Cell lines, plasmids, small interfering RNAs and transfection reagents

NPC cell lines (CNE2, HONE1, HNE2, HK1, CNE1, 5-8F, HNE1, 6-10B, and C666-1) and immortalized nasopharyngeal epithelial cell line NP69 were derived from the Cell Center of Central South University. All these cells were cultured on RPMI 1640 (Life Technologies, Grand Island, NY, USA) containing 10% fetal bovine serum (Gibco, Grand Island, NY, USA) at 37 °C and 5% CO_2_ and free of mycoplasma contamination.

The sequences of *circCAMSAP1* exons 2 and 3 were inserted into the pcDNA3.1(+) circRNA Mini Vector (kindly provided to us by professor Yong Li at Baylor College of Medicine, USA) to construct an overexpression vector. The SERPINH1-Flag, c-Myc-Flag, and SRSF10-Flag overexpression plasmids were purchased from Sino Biological Company (Beijing, China). The *SERPINH1* 3’UTR wild-type and mutant sequences were inserted into the pMIR-REPORT Luciferase miRNA Expression Reporter Vector (Ambion, TX, USA). The *CAMSAP1* promoter sequence was inserted into the PGL3 basic vector (E1751, Promega, USA).

The siRNAs that specifically targeted *circCAMSAP1*, SERPINH1, c-Myc, and SRSF10 were purchased from Gemma Gene Corporation (Shanghai, China), and the sequence is shown in Table S3.

Lipofectamine 3000 (Life Technologies, NY, USA) was used to transfect plasmids and Hiperfect (Qiagen, Hilden, Germany) was used to transfect siRNAs.

### RNA extraction and RT-qPCR

RNA extraction and RT-qPCR were performed according to the manufacturer’s instructions. The RT-qPCR was conducted using the HiScript cDNA synthesis kit (R323-01, Vazyme, China) and the SYBR RT-qPCR Master Mix (Q511-02, Vazyme, China). Primers are shown in Table S3. Relative gene expression was calculated by the comparative CT method (ΔΔCT), and *β-actin* was used as a housekeeping gene.

### Western blotting

Western blotting analysis was performed as described in previous studies [19]. Antibodies are shown in Table S4. Glyceraldehyde-3-phosphate dehydrogenase (GAPDH) was used as an endogenous control to normalize the protein loading.

### RNase R and actinomycin D treatment

RNase R enzyme (Thermo Fisher, Grand Island, NY, USA) digestion and actinomycin D (Sigma, Missouri, USA) treatment were used to detect the stability of RNA. For RNase R digestion, the total RNA extracted from NPC cells was added or not added to the RNase R enzyme (20 U/μL), incubated at 37 °C for 30 min and then at 70 °C for 10 min to inactivate the RNase R. This was followed by RT-qPCR detection. For the actinomycin D treatment, NPC cells were added with actinomycin D at a final concentration of 1 μg/mL at 0, 6, 12, 24 h, and RNA was collected for RT-qPCR analysis.

### Cycloheximide treatment

For the cycloheximide (CHX) treatment, NPC cells were added with CHX at a final concentration of 50 μg/mL at 0, 15, 30, 60 min, and protein was collected for western blotting analysis.

### In situ hybridization, fluorescence in situ hybridization, and immunohistochemistry

In situ hybridization (ISH) or fluorescence in situ hybridization (FISH) was carried out to detect *circCAMSAP1* expression in NPC tissues using digoxigenin-labeled probes as previously reported [19]. The Enhanced Sensitive ISH Detection kit I (POD) (MK1030, BOSTER, China) was used for ISH in NPC tissues. The ElivisionTM + Polyer HRP (Mouse/Rabbit) immunohistochemistry kit (Kit-9902, Maxim, China) was used to evaluate immunohistochemistry.

### Immunofluorescence

After 24 h of cell climbing, 4% paraformaldehyde was fixed for 30 min, 0.25% Triton X-100 was ruptured for 40 min, and 5% bovine serum albumin was blocked for 1 h. Cells were immuno-stained with antibodies overnight at 4 °C, washed and incubated with fluorescently labeled secondary antibodies (Thermo Fisher, Grand Island, NY, USA) at 37 °C for 40 min, and nuclei were stained with 6-diamidino-2-phenylindole (DAPI). A confocal laser scanning microscope (Perkin Elmer, Massachusetts, USA) was used for observation and imaging.

### Hematoxylin–eosin staining (H&E)

After the paraffin sections were dewaxed and watered with xylene and gradient alcohol, the nuclei were stained with hematoxylin solution (Beyotime, Shanghai, China), the eosin solution was used for staining cytoplasm (Beyotime, Shanghai, China), and the slides were mounted with neutral resin. Pictures were taken with an Olympus BX51 fluorescence microscope (Olympus, Japan).

### Wound-healing and transwell assay

Wound-healing assay was performed as previously reported [19]. An Olympus Digital Camera was used to capture the scratched area at 0, 12 and 24 h. The invasion assay was analyzed by transwell chambers with 8-μm pores (Millipore).

### MTT and flow cytometry

Eight hundred transfected cells were seeded into each well of a 96-well plate. After the cells adhered to the wall, 20 μl MTT (Beyotime, Shanghai, China) was added to each well for four additional hours. The 490 nm wavelength on the enzyme-linked instrument (Molecular Devices, California, USA) was used to detect the absorbance value. Flow cytometry was used to analyze the proliferation of NPC cells using propidium iodide staining.

### Liquid chromatography combined with tandem mass spectrometry (LC-MS/MS)

Protein identification by LC-MS/MS was performed using the previously reported method [19]. The peptide samples were diluted with 0.1% trifluoroacetic acid (TFA) (Sigma, Missouri, USA) and then transferred to the analysis chamber, where the Nano-LC LTQ Orbitrap ETD (Bruker Daltonic, Billerica, USA) was used for mass spectrometric analysis and identification. The Proteome Discoverer software (Thermo Scientific, Waltham, MA, USA), as well as the UniProtKB/Swiss-Prot database (release 2014_02), were used to identify the proteins.

### circRNA Immunoprecipitation (circRIP) assay

The biotin-labeled *circCAMSAP1* probe was used for RNA pull-down. The *circCAMSAP1* overexpression plasmid and probe were transfected into NPC cells. Cells were incubated with streptavidin Dynabeads (M-280, Invitrogen) at 4 °C. Immunoprecipitated RNA was isolated with TRIzol reagent and analyzed by RT-qPCR. The primers and probe sequences used are shown in Table S3.

### RNA pull-down assay and co-immunoprecipitation

RNA pull-down assay was performed using a magnetic RNA protein pull-down kit (20164, Thermo Scientific) according to the manufacturer’s instructions. Cell lysates were incubated with the streptavidin Dynabeads or antibodies, and the enriched proteins were denatured at high temperatures, then examined using western blotting.

### Luciferase reporter assays and chromatin immunoprecipitation (ChIP)

The Dual-Luciferase® Reporter Assay System (E2980, Promega, USA) was used according to the manufacturer’s instructions.

ChIP analysis was performed using the Pierce™ Magnetic ChIP Kit (26157, Thermo Scientific, USA). The c-Myc antibody was used to enrich the chromatin fragments in NPC cells, and the enriched chromatin fragments were analyzed by RT-qPCR. RT-qPCR primers were designed according to the binding sites (Table S3).

### RNA binding protein immunoprecipitation (RIP) assay

RIP assay was performed with the RIP kit (Millipore, Billerica, MA, USA) according to the manufacturer’s instructions. See Table S3 for detection primers.

### Animal testing

First, CNE2 (2 × 10^6^) transfected with the *circCAMSAP1* overexpression vector, *circCAMSAP1* siRNA1, or the empty vector and scrambled siRNA were injected through the tail vein of nude mice (5 mice per group). After 2 months, the nude mice were sacrificed by cervical dislocation. Lung tissues were collected, and pulmonary nodules were counted. ISH, H&E staining, and immunohistochemistry were performed to examine the expression of *circCAMSAP1*, SERPINH1 and c-Myc.

Then, cells were subcutaneously injected into the armpit of nude mice. The size of the subcutaneous tumor nodule was measured every 3 days. After 27 days, the nude mice were and the subcutaneous tumor size was measured. Tumor volume, size, and weight and the body weight of the nude mice were measured. ISH, H&E staining, or immunohistochemistry were performed to examine the expression of *circCAMSAP1*, SERPINH1, and c-Myc. All animal studies were approved by the Ethics Committee of the Xiangya Hospital, Central South University.

### Statistical analysis

The GraphPad Prism 8 (https://www.graphpad.com/scientific-software/prism/) was used for all statistical analyses in this study. The unpaired t-test was performed on each group of data, and a *p*-value of < 0.05 was considered to show a significant difference. Image Pro Plus v.6.0 (Media Cybernetics, Inc. Rockville, USA) was used to analyze the images from the wound-healing and transwell assays. Image J software (https://imagej.nih.gov/ij/download.html) was used to analyze the images from western blotting.

## Results

### *circCAMSAP1* is highly expressed in NPC

To evaluate the role of circRNAs in the development of NPC, the high-throughput RNA sequencing (GSE68799) of NPC including 41 NPC tissues and 4 chronically inflamed nasopharyngeal epithelium tissues was reanalyzed. A total of 568 circRNAs that are not expressed in normal nasopharyngeal tissues but are expressed in NPC tissues were obtained according to their spliced RPM values. The top 20 circRNAs expressed in NPC tissues were selected for subsequent analysis by Sanger sequencing (Table S5). Notably, *hsa_circ_0001900*, which is located on chromosome 9 (chr9:138773478–138774923), was not reported in NPC (Fig. S1A).

**Fig. 1:**
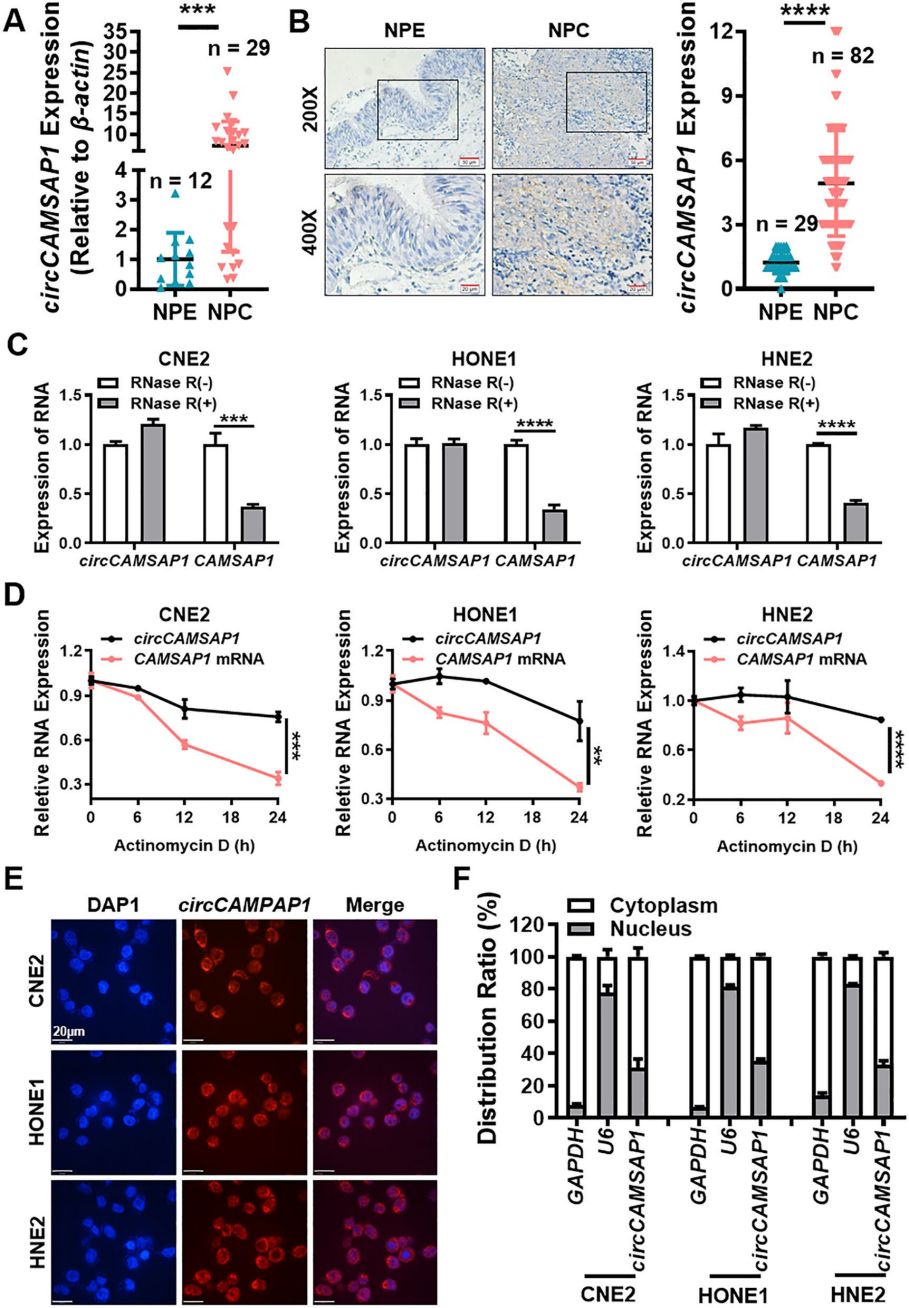
*circCAMSAP1* is highly expressed in NPC. **A** The expression of *circCAMSAP1* was measured in 29 NPC tissues and 12 non-cancerous NPE tissue samples by RT-qPCR. *β-actin* was used as an internal reference. NPE, nasopharyngeal epithelium; NPC, nasopharyngeal carcinoma. ***, *p* < 0.001. **B** Representative images of *circCAMSAP1* in 82 NPC tissues and 29 adjacent NPE tissues by in situ hybridization. × 200, scale bar = 50 μm; × 400, scale bar = 20 μm. The statistical data was shown in the right panel. ****, *p* < 0.0001. **C** The stability of *circCAMSAP1* was detected in three RNase R-treated NPC cells by RT-qPCR. *CAMSAP1* mRNA was used as a negative control. ***, *p* < 0.001; ****, *p* < 0.0001. **D** The stability of *circCAMSAP1* was examined in NPC cells after treating with actinomycin D for 0, 6, 12 and 24 h. The relative RNA levels of *circCAMSAP1* and *CAMSAP1* mRNA in NPC cells were measured by RT-qPCR. **, *p* < 0.01; ***, *p* < 0.001; ****, *p* < 0.0001. **E** Intracellular localization of *circCAMSAP1* (red) in three NPC cells, as determined by fluorescence in situ hybridization using a digoxigenin-labeled *circCAMSAP1* probe. Nuclei were stained with DAPI (blue). Scale bar = 20 μm. **F** The cellular localization of *circCAMSAP1* was examined in three NPC cells by nuclear and cytoplasmic separation test. *GAPDH* and *U6* were used as positive controls for cytoplasm and nucleus, respectively

To verify the expression of *circCAMSAP1* in NPC, 29 NPC tissues and 12 chronic rhinitis epithelial tissues (as normal controls) were used for RT-qPCR. The results showed that *circCAMSAP1* was expressed significantly more in the 29 NPC samples (Fig. 1A) than in the 12 non-cancer nasopharyngeal epithelial (NPE) tissues. Moreover, the expression of *circCAMSAP1* was significantly correlated with a high T stage (*p* < 0.05) in NPC patients. However, there was no significant correlation between expression of *circCAMSAP1* and N stage classification (Fig. S1B).

Then, the high expression of *circCAMSAP1* in NPC tissue was further confirmed in 82 NPC and 29 non-cancer NPE paraffin sections using ISH. The data showed that *circCAMSAP1* was expressed significantly more in NPC tissue samples (Fig. 1B) and the high expression of *circCAMSAP1* was significantly correlated with high T stage (*p* < 0.01), N stage (*p* < 0.0001), and M stage (*p* < 0.001) classification in NPC patients (Fig. S1C). Furthermore, *circCAMSAP1* was also found to be more highly expressed in NPC cell lines than in immortalized NPE cell line NP69 by RT-qPCR (Fig. S1D). Hence, *circCAMSAP1* expression could serve as a prognostic biomarker for NPC.

The reverse splicing of exons 2 and 3 of the *CAMSAP1* gene produces *circCAMSAP1* as seen in the circBase database (Fig. S1E). Moreover, circRNAs are more stable than intracellular mRNA. As shown in Fig. 1C, resistance to digestion by RNase R exonuclease confirmed that *circCAMSAP1* is indeed circular. The results of actinomycin D assays revealed that the half-life of the *circCAMSAP1* transcript is longer than the half-life of the *CAMSAP1* mRNA, indicating that *circCAMSAP1* is more stable than the linear *CAMSAP1* transcript (Fig. 1D). RNA FISH analysis and RNA nuclear and cytoplasmic isolation showed that *circCAMSAP1* was distributed predominantly in the cytoplasm of NPC (Fig. 1E, F). Hence, *circCAMSAP1* was selected for the evaluation of NPC tumorigenesis in the present study.

### *circCAMSAP1* promotes the metastasis and proliferation of NPC cells in vitro

**Fig. 2:**
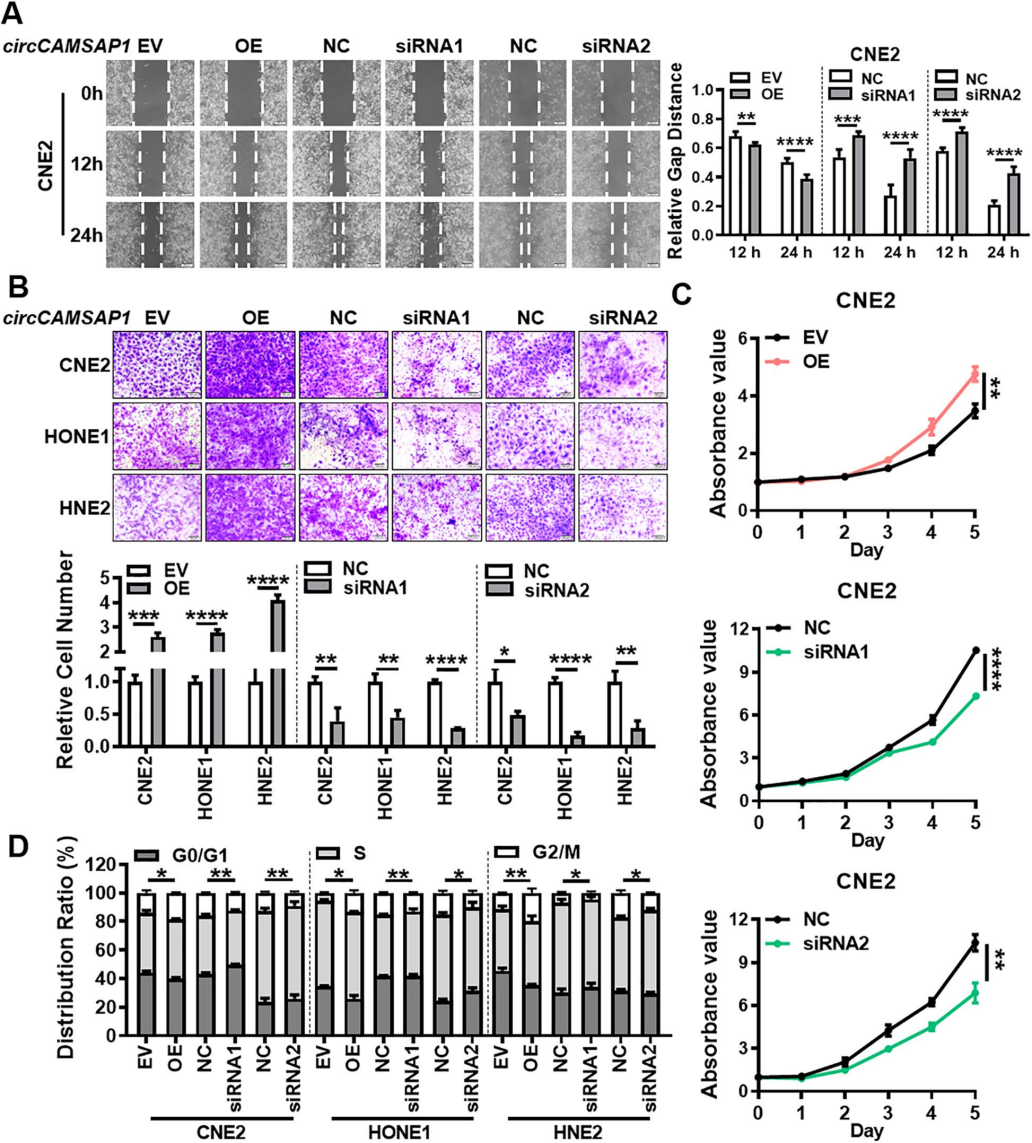
*circCAMSAP1* promotes proliferation, migration, and invasion of NPC cells in vitro. Wound-healing assay (**A**), transwell invasion (**B**), MTT assays (**C**), and flow cytometry analysis of cell cycle distribution (**D**) in the empty vector, the *circCAMSAP1* overexpression vector, two *circCAMSAP1* siRNAs, and scramble siRNAs transfected NPC cells. EV, empty vector; OE, the overexpression vector; NC, scrambled negative control; Data are shown as means ± standard deviation (SD). *, *p* < 0.05; **, *p* < 0.01; ***, *p* < 0.001; ****, *p* < 0.0001

To investigate the potential functions of *circCAMSAP1* in regulating the biological behavior of NPC cells, two siRNAs for *circCAMSAP1* were first designed to target the back-splice site of *circCAMSAP1*. The *circCAMSAP1* overexpression vector was also constructed. RT-qPCR confirmed the overexpression or knockdown efficiency of the *circCAMSAP1* overexpression vector or two siRNAs in CNE2, HONE1, and HNE2 cells after transfection (Fig. S2A). Wound-healing and transwell assays demonstrated that *circCAMSAP1* upregulation significantly enhances migration and invasion of CNE2, HONE1, and HNE2 cells, whereas *circCAMSAP1* downregulation inhibits cell migration and invasion (Fig. 2A-B, Fig. S2B). MTT assays showed that *circCAMSAP1* overexpression markedly increases proliferation and viability, while *circCAMSAP1* knockdown produces the opposite effect (Fig. 2C, Fig. S2C). Cell cycle analysis revealed that overexpression of *circCAMSAP1* leads to increased rates of NPC cells in G2/M phases, suggesting that upregulation of *circCAMSAP1* results in G2/M arrest in NPC cells (Fig. 2D, Fig. S2D). These results show that *circCAMSAP1* promotes the development of NPC by promoting migration, invasion, and proliferation of NPC cells.

### *circCAMSAP1* promotes the metastasis and proliferation of NPC cells in vivo

**Fig. 3:**
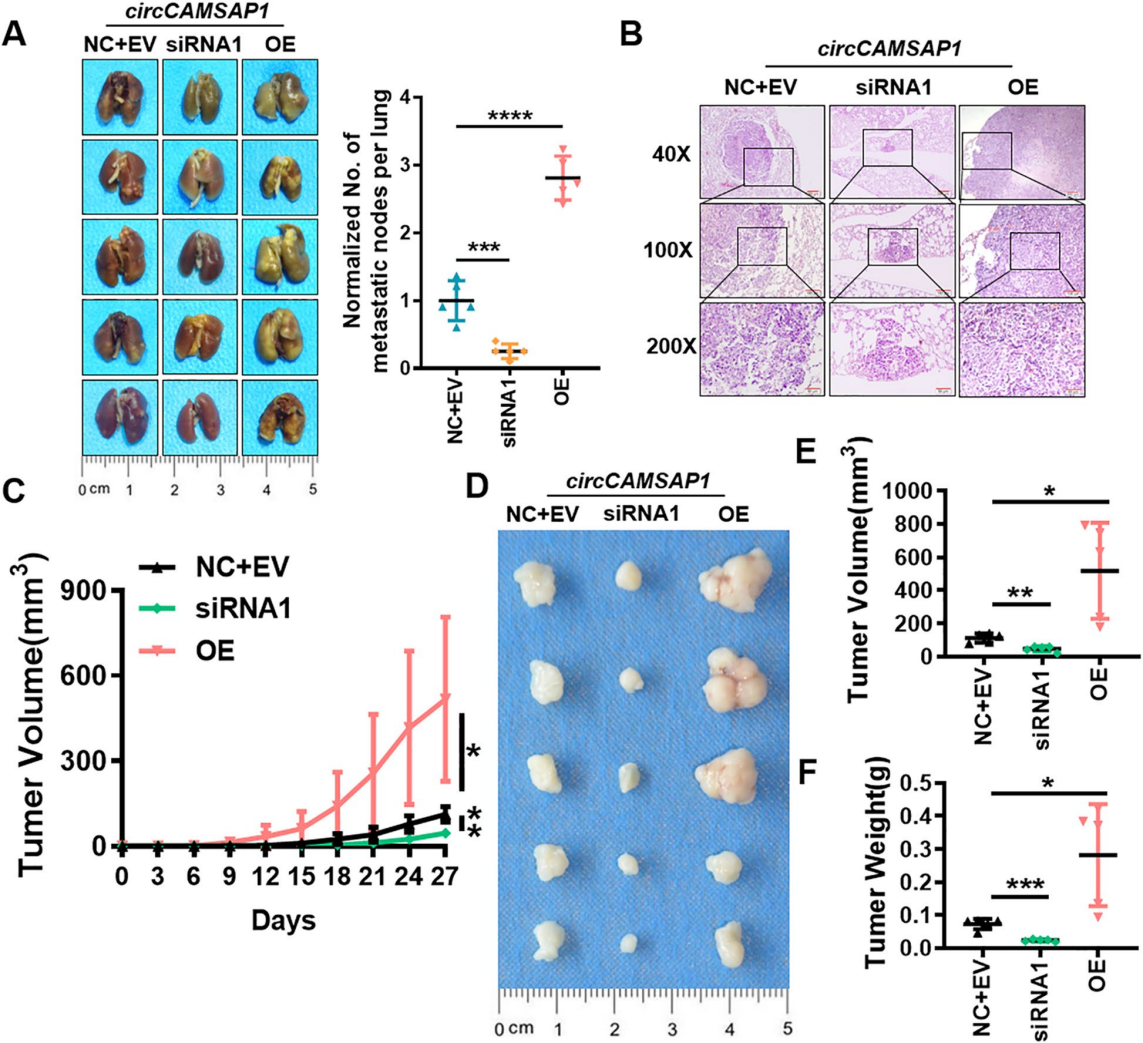
*circCAMSAP1* promotes proliferation and metastasis of NPC cells in vivo*.* **A** Representative images of nude mice lung tissues after tail vein injection of NPC cells. Mice were injected with NPC cells after overexpression or knockdown of *circCAMSAP1* (Left). The number of nodules on the surface of lung tissues were counted and analyzed (Right). *n* = 5; ***, *p* < 0.001; ****, *p* < 0.0001. **B** Representative images of hematoxylin and eosin-stained metastatic lung nodules after overexpression or knockdown of *circCAMSAP1*. × 40, scale bar = 200 μm; × 100, scale bar = 100 μm; × 200, scale bar = 50 μm. **C** Growth curve of subcutaneous tumor volume in nude mice. Nude mice were injected with NPC cells subcutaneously in the armpit after overexpression or knockdown of *circCAMSAP1*. *n* = 5; *, *p* < 0.05; **, *p* < 0.01. **D-F** Image of subcutaneous tumor tissues in nude mice (**D**), tumor volume (**E**) and tumor weights (**F**) show that *circCAMSAP1* promoted CNE2 xenograft growth 27 days after subcutaneous injection. *n* = 5; *, *p* < 0.05; **, *p* < 0.01; ***, *p* < 0.001

To investigate the cancer-promoting role of *circCAMSAP1* in vivo, we further evaluated the effect of *circCAMSAP1* silencing and overexpression on NPC progression in xenografts. The nude mouse lung metastasis model showed that the number of intrahepatic metastatic nodules was much lower in the mice inoculated with the si*circCAMSAP1*/CNE2 cells than mice transplanted with the negative control/CNE2 cells. While mice transplanted with the *circCAMSAP1*/CNE2 cells showed more intrahepatic metastatic nodules than that with the negative control/CNE2 cells (Fig. 3A-B).

The subcutaneous tumor model of nude mice was constructed by injecting CNE2 into the armpit of nude mice. The data showed that the volume and weight of the tumors formed in the *circCAMSAP1* knockdown group were significantly lower than those of the tumors in the group treated with the negative control, while *circCAMSAP1* overexpression produced the opposite effect (Fig. 3C-F). Therefore, we conclude that *circCAMSAP1* may act as an oncogene and promote NPC growth and metastasis.

### *circCAMSAP1* stabilizes the expression of *SERPINH1* by binding to its 3’UTR

High-throughput mass spectrometry was carried out to explore the function of *circCAMSAP1* in NPC and identify the possible signaling pathway by which *circCAMSAP1* promotes proliferation, migration, and invasion in CNE2 cells after overexpression or knockdown of *circCAMSAP1*. A total of 2415 protein molecules were analyzed by mass spectrometry, 97 protein molecules were positively associated with *circCAMSAP1* expression, while 146 protein molecules were negatively associated with *circCAMSAP1* expression (Table S6, Fig. S3A). To elucidate the regulatory mechanism of the influence of *circCAMSAP1* on signaling, the expression of the 97 protein molecules positively associated with *circCAMSAP1* was analyzed from the NPC microarray data (GSE12452, GSE53819). Among them, SERPINH1, a molecular chaperone which has been reported to be closely related to tumor proliferation and metastasis [21] was highly expressed in NPC tissues (Fig. S3B). Our RT-qPCR results also showed that *SERPINH1* mRNA was highly expressed in the 29 NPC tissues and correlated with the expression of *circCAMSAP1* (Fig. S3C-D).

**Fig. 4:**
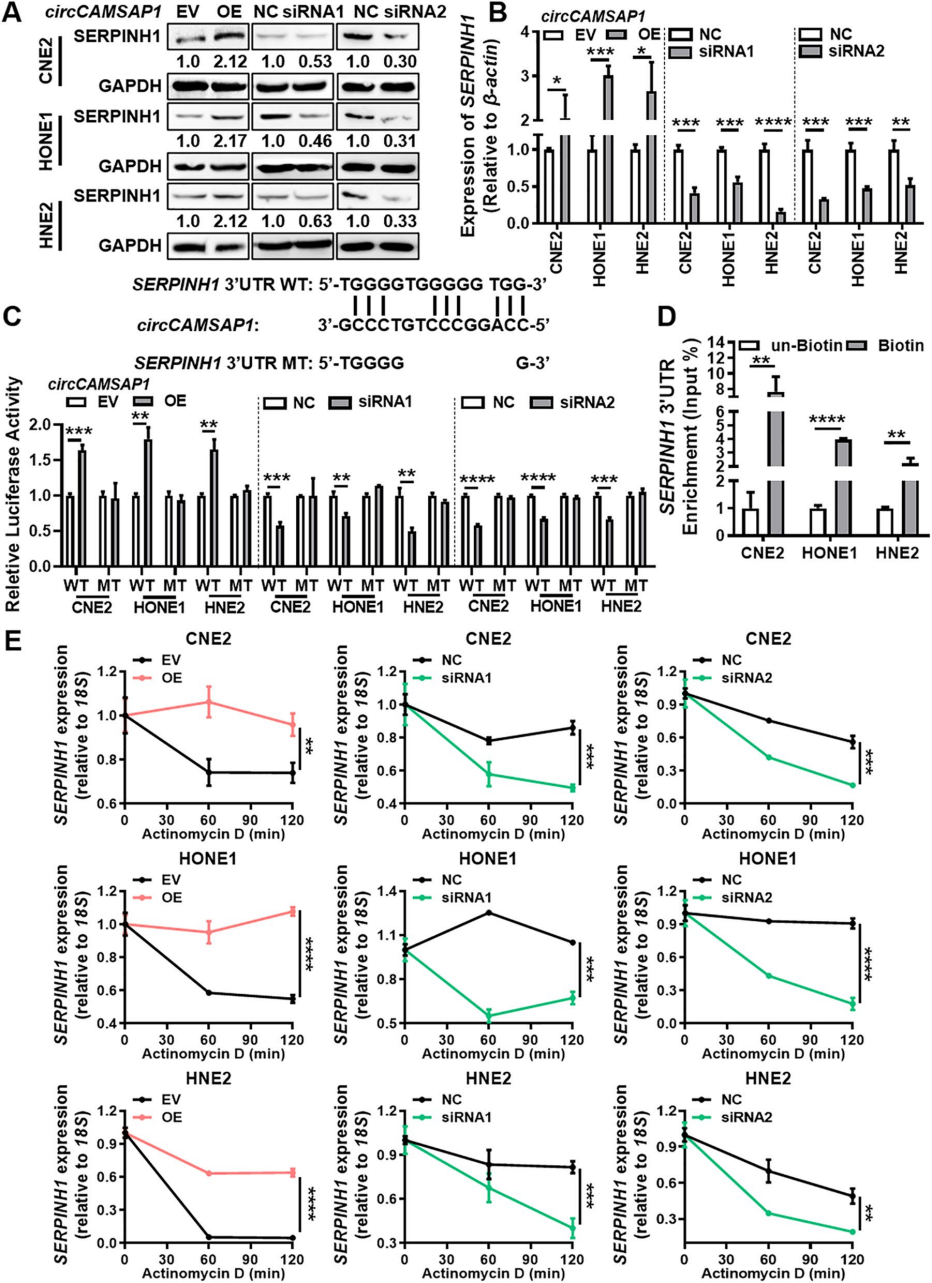
*circCAMSAP1* stabilizes the expression of *SERPINH1* by binding to its 3’UTR. **A** Western blotting analysis of SERPINH1 expression in three NPC cell lines after overexpression and knockdown of *circCAMSAP1*. **B** RT-qPCR analysis of *SERPINH1* expression in three NPC cell lines after overexpression and knockdown of *circCAMSAP1*. *, *p* < 0.05; **, *p* < 0.01; ***, *p* < 0.001; ****, *p* < 0.0001. **C** The luciferase reporter gene activity was analyzed in three NPC cell lines after overexpression or knockdown of *circCAMSAP1*. The wild-type (WT) or mutant (MT) of the *SERPINH1* 3’UTR were constructed and transfected into NPC cells. **, *p* < 0.01; ***, *p* < 0.001; ****, *p* < 0.0001. **D** The circRIP assay was used to detect the binding of *circCAMSAP1* to the 3’UTR of *SERPINH1* mRNA. **, *p* < 0.01; ****, *p* < 0.0001. **E** The stability of *SERPINH1* mRNA was measured in NPC cells after overexpression or knockdown of *circCAMSAP1*. Cells were treated with actinomycin D for 0, 60, 120 min and RT-qPCR was used to measure the expression of *SERPINH1* mRNA. **, *p* < 0.01; ***, *p* < 0.001; ****, *p* < 0.0001

To depict other mechanisms that are responsible for the relationship between *circCAMSAP1* and SERPINH1 in NPC, western blotting analysis, and RT-qPCR were performed in NPC cells after overexpression or knockdown of *circCAMSAP1*. The data showed that *circCAMSAP1* can positively regulate the protein and mRNA expression of SERPINH1 (Fig. 4A-B). RNA pull-down showed that *circCAMSAP1* is unable to bind directly to the SERPINH1 protein (Fig. S3E). Additionally, bioinformatics analysis using the RNA22 software showed that *circCAMSAP1* has a continuous binding sequence to the 3’UTR of the *SERPINH1* mRNA (Fig. S3F). In eukaryotic cells, the 3’UTR of mRNAs plays a role in maintaining mRNA stability [13]. Thus, the binding of *circCAMSAP1* to the 3’UTR of *SERPINH1* mRNA may delay the degradation of *SERPINH1* mRNA by nucleic acid exonucleases, ultimately leading to elevated *SERPINH1* expression. The dual-luciferase reporter gene assay showed that *circCAMSAP1* could regulate the luciferase activity of the *SERPINH1* 3’UTR (Fig. 4C). The circRIP assay confirmed that *circCAMSAP1* binds to the 3’UTR of *SERPINH1* mRNA in NPC cells (Fig. 4D), and overexpression of *circCAMSAP1* enhances the stability of *SERPINH1*, while knockdown of *circCAMSAP1* gives the opposite result in NPC cells after actinomycin D treatment (Fig. 4E).

**Fig. 5:**
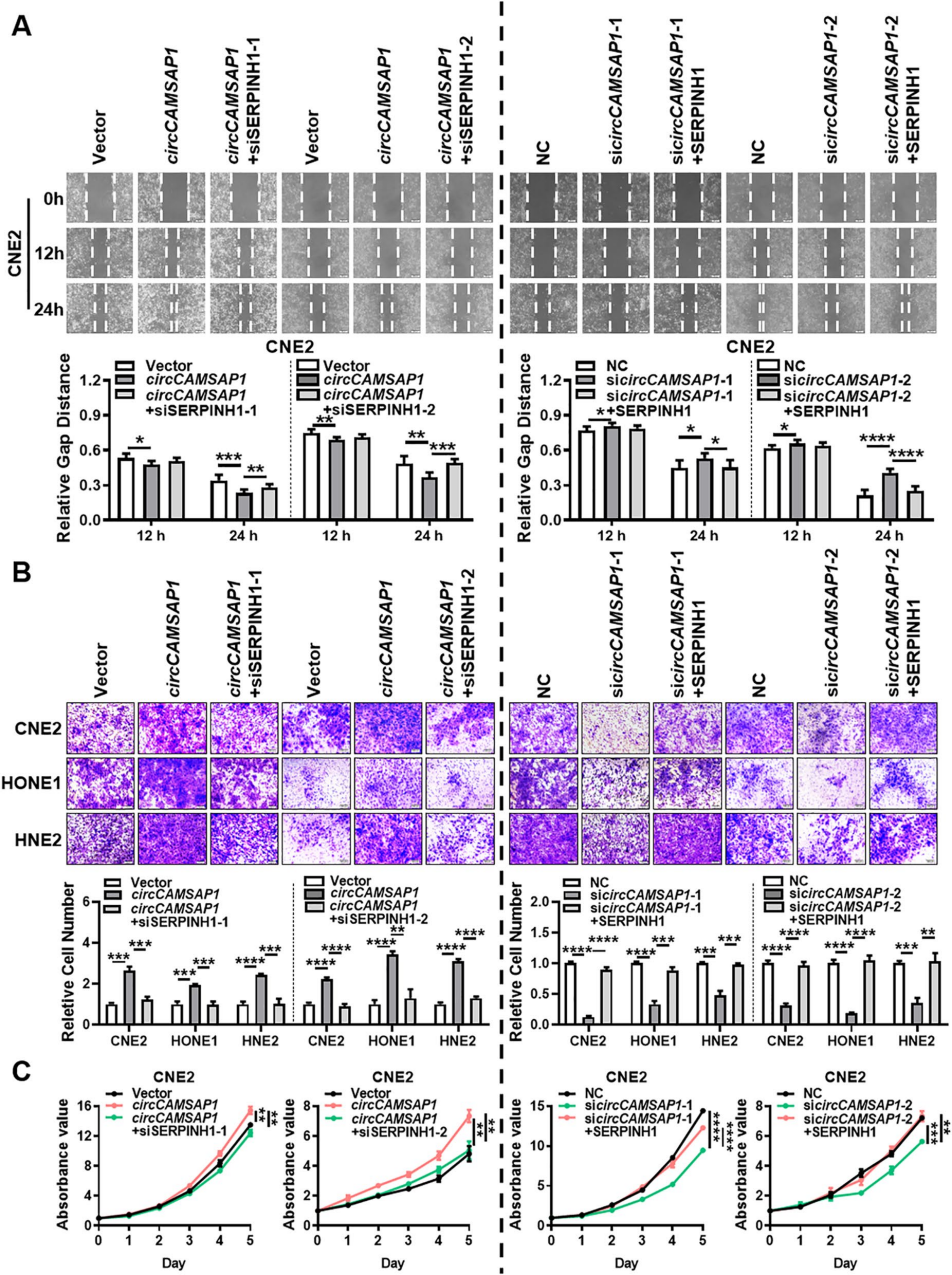
*circCAMSAP1* promotes proliferation, migration, and invasion of NPC through SERPINH1. Wound-healing assay (**A**), transwell invasion (**B**), and MTT assays (**C**) in NPC cells after knockdown or overexpression of *circCAMSAP1* and SERPINH1. Data are shown as means ± SD. *, *p* < 0.05; **, *p* < 0.01; ***, *p* < 0.001; ****, *p* < 0.0001

To illustrate whether SERPINH1 is critical for *circCAMSAP1*-induced growth, migration, and invasion of NPC cells, we overexpressed or knocked down *circCAMSAP1* or SERPINH1 in NPC cells. The results revealed that overexpression of SERPINH1 promotes NPC cell growth, migration, and invasion, while knockdown of SERPINH1 has the opposite effect (Fig. S4). Specifically, knockdown of SERPINH1 significantly alleviates the promotive effect of *circCAMSAP1* overexpression on proliferation (Fig. 5C, Fig. S5B), migration (Fig.5A, Fig. S5A) and invasion (Fig. 5B) of NPC cells. Additionally, overexpression of SERPINH1 partially restored the inhibitory effect of *circCAMSAP1* knockdown on proliferation (Fig. 5C, Fig. S5B), migration (Fig.5A, Fig. S5A), and invasion (Fig. 5B) of NPC cells. Collectively, these data demonstrated that *circCAMSAP1* increases the expression of SERPINH1 and promotes cell proliferation and metastasis of NPC cells by binding to the 3’UTR of *SERPINH1*.

### SERPINH1 inhibits c-Myc’s ubiquitination-degradation

**Fig. 6:**
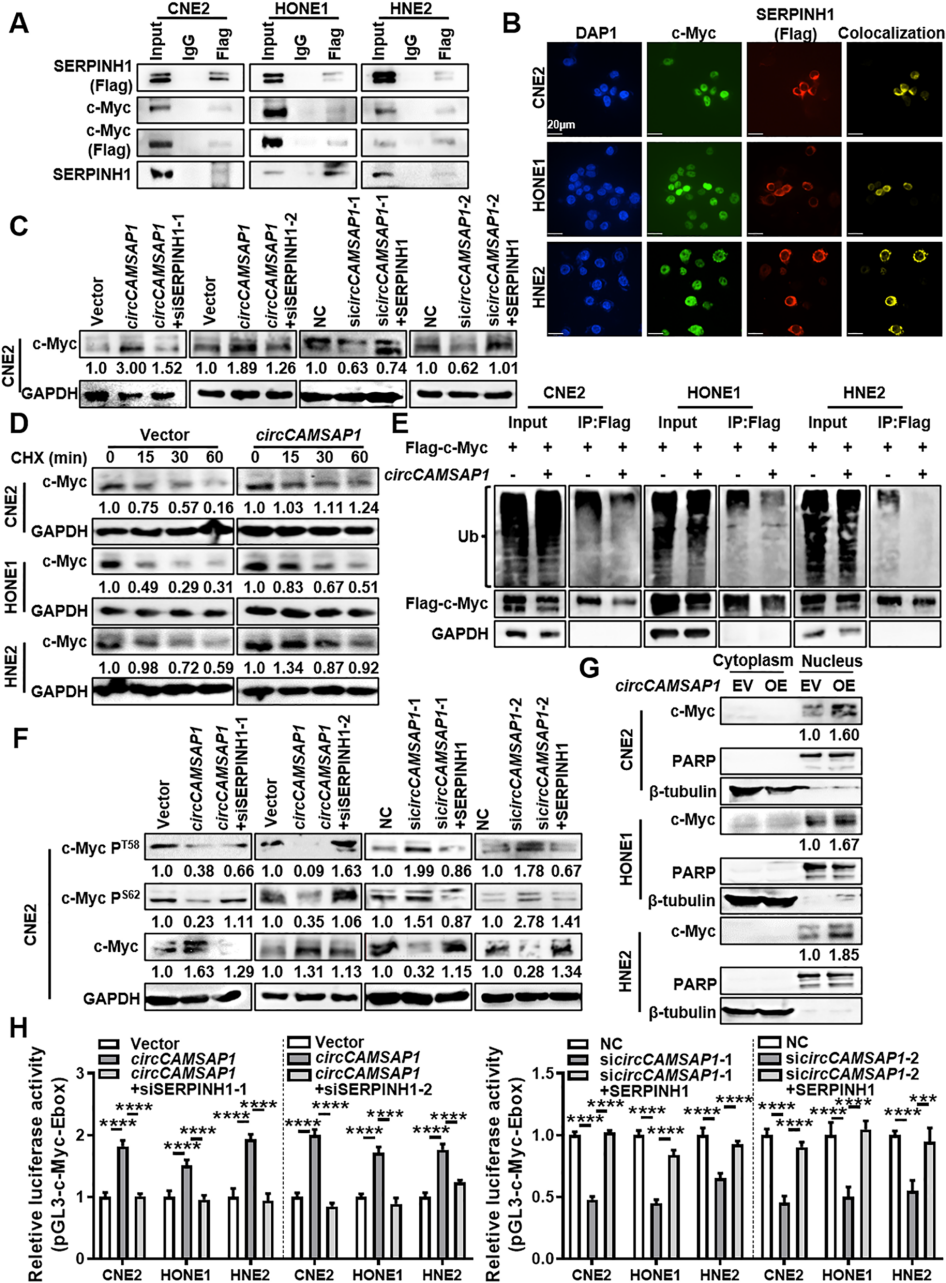
SERPINH1 binds to c-Myc and inhibits its ubiquitination-degradation. **A** Co-IP assay was shown the interaction between SERPINH1 and c-Myc in three NPC cell lines. **B** Co-localization of SERPINH1 and c-Myc protein in NPC cells was determined by immunofluorescence. Scale bars = 20 μm. **C** Western blotting analysis of c-Myc expression in CNE2 cells after overexpression of *circCAMSAP1* and knockdown of SERPINH1 or *circCAMSAP1* or overexpression of SERPINH1. **D** Western blotting analysis of the c-Myc protein stability in NPC cells after treatment with cycloheximide (CHX). **E** Western blotting analysis of the ubiquitination of c-Myc protein after overexpression of *circCAMSAP1* in NPC cells. **F** The phosphorylation level of c-Myc T58 and S62 phosphorylation sites was measured by western blotting in CNE2 cells after overexpression of *circCAMSAP1* and knockdown of SERPINH1, or knockdown of *circCAMSAP1* and overexpression of SERPINH1. **G** The nuclear c-Myc expression was examined in NPC cells after overexpression of *circCAMSAP1* in CNE2 cells by western blotting. **H** The transcript activity of c-Myc was measured in NPC cells after overexpression of *circCAMSAP1* and knockdown of SERPINH1, or knockdown of *circCAMSAP1* and overexpression of SERPINH1. ***, *p* < 0.001; ****, *p* < 0.0001

To identify the possible function of SERPINH1 on the proliferation, migration, and invasion of NPC cells regulated by *circCAMSAP1*, four different interaction databases (HitPredict, BioGRID, UniHI, and GeneMANIA) were used to identify the proteins that interact with SERPINH1. We then selected c-Myc because it was the only protein predicted to bind to SERPINH1 by all four interaction databases and it plays an important role in tumorigenesis and development [22]. Immunoprecipitation analysis (Fig. 6A) showed that SERPINH1 can bind to c-Myc in NPC cells and immunofluorescence showed the co-localization between c-Myc and SERPINH1 (Fig. 6B). Western blotting showed that SERPINH1 could positively regulate the expression of c-Myc (Fig. S6A).

To identify whether *circCAMSAP1* participates in the interaction of SERPINH1 with c-Myc, *circCAMSAP1* or SERPINH1 was overexpressed or knocked down in NPC cells. Western blotting showed that knockdown of SERPINH1 could inhibit the upregulation of c-Myc expression caused by overexpression of *circCAMSAP1*, while overexpression of SERPINH1 could improve downregulation of c-Myc expression caused by *circCAMSAP1* (Fig. 6C, Fig. S6B). RT-qPCR assays further showed that *circCAMSAP1* and SERPINH1 had no effect on the mRNA expression level of *c-Myc* (Fig. S6C). This suggests that the effect of *circCAMSAP1* and SERPINH1 on the expression of c-Myc may be at the post-translational level.

We then treated NPC cells with cycloheximide (CHX) to identify the effect of *circCAMSAP1* and SERPINH1 on the stability of the c-Myc protein in NPC cells. The results showed that *circCAMSAP1* and SERPINH1 could inhibit the degradation of the c-Myc protein (Fig. 6D, Fig. S7A).

A common degradation pathway of c-Myc is through ubiquitination. The degree of ubiquitination of the protein is estimated by the rate of binding of c-Myc to the ubiquitinated protein [23]. When NPC cells are treated with MG132, a proteasome inhibitor, overexpression of *circCAMSAP1* inhibits c-Myc ubiquitination (Fig. 6E), while inhibition of SERPINH1 promotes c-Myc ubiquitination (Fig. S7B). The change of c-Myc phosphorylation level is tightly correlated to the ubiquitination-degradation of c-Myc [24]. The phosphorylation level of c-Myc at the T58 and S62 sites, and the results showed that overexpression of SERPINH1 could inhibit the phosphorylation of c-Myc, while knockdown of SERPINH1 has the opposite result (Fig. S7C). Knockdown of SERPINH1 can promote the downregulation of c-Myc phosphorylation caused by overexpression of *circCAMSAP1*, while overexpression of SERPINH1 can inhibit the upregulation of c-Myc phosphorylation caused by *circCAMSAP1* knockdown (Fig. 6F, Fig. S7D).

As a transcription factor, c-Myc regulates the expression of many proteins, plays an important role in cell proliferation, cycle regulation, invasion, and metastasis, and affects the progression of many tumors [25]. Therefore, we tested whether *circCAMSAP1* and SERPINH1 can increase the abundance of c-Myc expression in the nucleus through the nuclear-plasmin separation protein analysis. The results showed that *circCAMSAP1* and SERPINH1 could increase c-Myc expression in the nucleus (Fig. 6G, Fig. S8A). The c-Myc transcription factor usually works with a promoter containing E-box (5′-CACGTG-3′), so we used a luciferase reporter plasmid containing three E-boxes to explore the effects of *circCAMSAP1* and SERPINH1 on the transcriptional activity of c-Myc. The results showed that overexpression of SERPINH1 could promote the transcriptional activity of c-Myc in NPC cells, while knockdown of SERPINH1 has the opposite result (Fig. S8B). Furthermore, knockdown of SERPINH1 could inhibit the promotion of c-Myc transcriptional activity by *circCAMSAP1*, while overexpression of SERPINH1 could decrease c-Myc transcriptional activity caused by knockdown of *circCAMSAP1* (Fig. 6H).

The expression of *circCAMSAP1*, SERPINH1 and c-Myc in nude mice lung metastases and subcutaneous tumors was also analyzed. ISH and immunohistochemistry showed that *circCAMSAP1*, SERPINH1, and c-Myc were highly expressed and positively correlated in lung metastases and subcutaneous tumors in the overexpression group, but low in the knockdown group, compared with the negative control group (Fig. S9).

### c-Myc combined with SRSF10 promotes *circCAMSAP1* expression

**Fig. 7:**
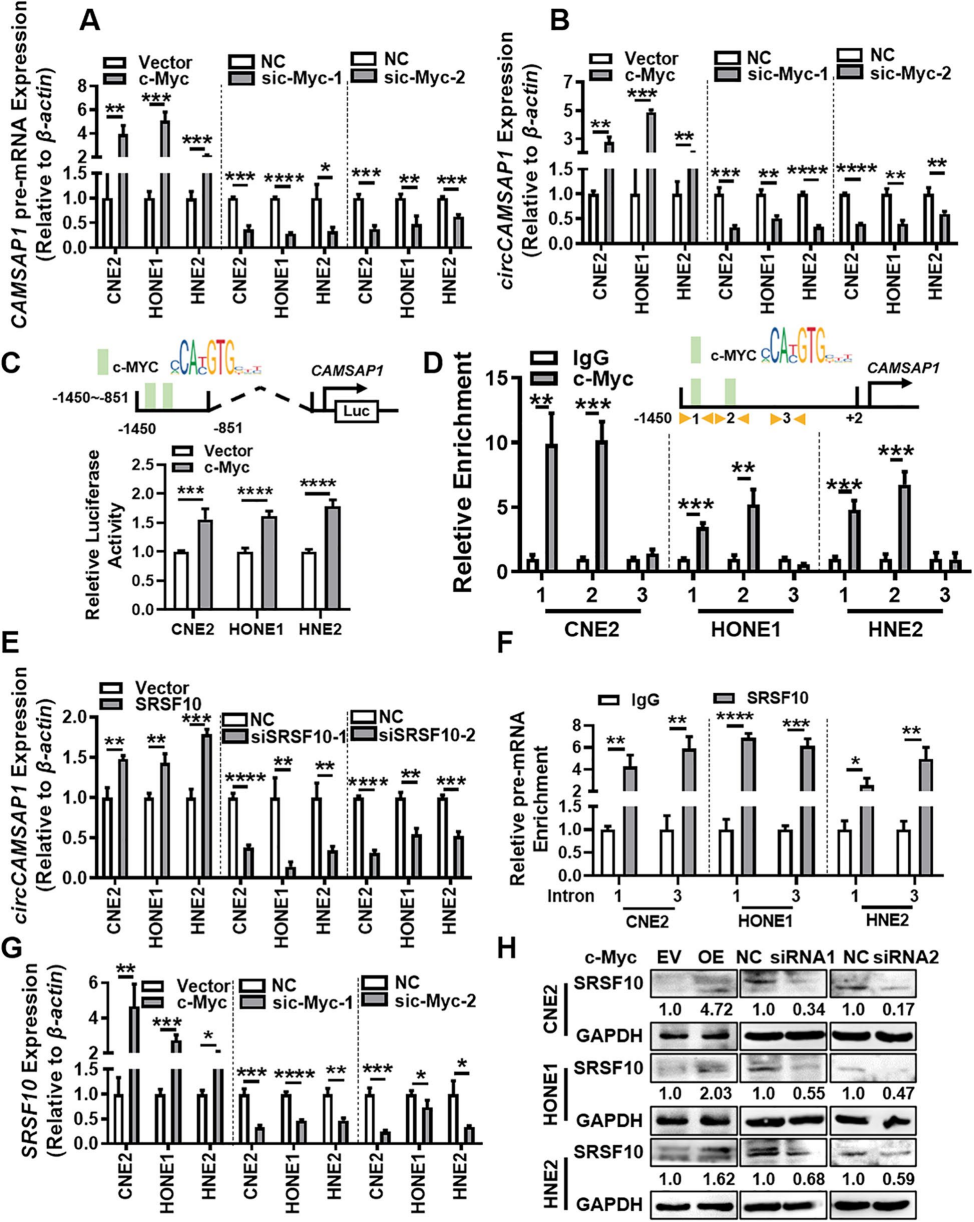
c-Myc combines with SRSF10 to promote the expression of *circCAMSAP1.* **A** The expression of *CAMSAP1* pre-mRNA was examined by RT-qPCR in NPC cells after overexpression or knockdown of c-Myc. *, *p* < 0.05; **, *p* < 0.01; ***, *p* < 0.001; ****, *p* < 0.0001. **B** The expression of *circCAMSAP1* was examined by RT-qPCR in NPC cells after overexpression or knockdown of c-Myc. **, *p* < 0.01; ***, *p* < 0.001; ****, *p* < 0.0001. **C** The luciferase reporter gene activity of the *CAMSAP1* promoter was analyzed in NPC cells after overexpression of c-Myc. ***, *p* < 0.001; ****, *p* < 0.0001. **D** The binding of transcription factor c-Myc to the *CAMSAP1* promoter detected by ChIP assay. Two sites on the *CAMSAP1* promoter were selected according to the c-Myc binding sites (site 1 and site 2) using the Jasper and PROMO software. A non-binding region (site 3) was used as a negative control. **, *p* < 0.01; ***, *p* < 0.001. **E** The expression of *circCAMSAP1* was examined by RT-qPCR in NPC cells after overexpression or knockdown of SRSF10. **, *p* < 0.01; ***, *p* < 0.001; ****, *p* < 0.0001. **F** SRSF10 bound to the flanking intron sequence of *circCAMSAP1* detected by RIP assay. Intron 1 and intron 3 were selected for RIP experiments using the SRSF10 antibody and referred to the SRSF10 binding site of *CAMSAP1* pre-mRNA. *, *p* < 0.05; **, *p* < 0.01; ***, *p* < 0.001; ****, *p* < 0.0001. **G** The expression of *SRSF10* was examined by RT-qPCR in NPC cells after knockdown of c-Myc. *, *p* < 0.05; **, *p* < 0.01; ***, *p* < 0.001; ****, *p* < 0.0001. **H** The SRSF10 expression in NPC cells after knockdown of c-Myc examined by western blotting

To explore the mechanism of high expression of *circCAMSAP1* in NPC tissues, the expression of *circCAMSAP1* was examined in NPC cells after overexpressing or knocking down c-Myc (Fig. S10A-B). It was found that c-Myc positively regulates the expression of *circCAMSAP1*, *CAMSAP1* pre-mRNA, and *CAMSAP1* mRNA (Fig. 7A-B, Fig. S10C) in NPC cells. The expression of *CAMSAP1* mRNA levels in NPC tissues may be up-regulated (Fig. S10D). Bioinformatics software (PROMO and JASPER databases) showed that c-Myc could bind near the positions -1391 bp and -1211 bp of the *CAMSAP1* promoter (Fig. S10E). The luciferase reporter confirmed that c-Myc binds to the *CAMSAP1* promoter region (-1450 bp- -851 bp) (Fig. 7C). ChIP analysis further showed that c-Myc mainly binds to nucleotide -1391 bp and-1211 bp in the *CAMSAP1* promoter (Fig. 7D).

CircRNA is produced by alternative splicing of pre-mRNA [9]. Splicing factors act on the RNA surrounding the circRNA sequence on the pre-mRNA strand to promote the occurrence of splicing and increase the expression of circRNA [26]. We used the RBPmap database to predict splicing factors that may bind to the introns flanking exons 2 and 3 of *CAMSAP1* pre-mRNA. SRSF10 had been predicted binding to the introns flanking exons 2 and 3 of *CAMSAP1* pre-mRNA and was also highly expressed in NPC cells (Fig. S10F). When SRSF10 is overexpression or knocked down in NPC cells (Fig. S10G), the expression of *circCAMSAP1* is increased or decreased (Fig. 7E). RIP assay confirmed the binding of SRSF10 to the flanking introns of *circCAMSAP1* (Fig. 7F). Moreover, c-Myc could also promote the expression of SRSF10 (Fig. 7G-H). Taken together, these results indicate that c-Myc is involved in the transcription and formation of *circCAMSAP1*, cooperating with SRSF10. The positive feedback of the *circCAMSAP1*-SERPINH1-c-Myc axis may play crucial roles in NPC progression.

## Discussion

The local recurrence and distant metastasis of NPC are major obstacles in the clinical treatment of NPC. Future research should be aimed to explore the molecular mechanism of NPC metastasis and proliferation and find the molecules active in the mechanism [27, 28]. Recent studies have shown that non-coding RNA, including microRNA [29], long non-coding RNA [30], and circRNA [31] play an important role in the occurrence and development of NPC. Among them, *circCRIM1* [32], *circTGFBR2*, *circHIPK3* [33] and *circ_0046263* [34] play an important role in the metastasis and proliferation of NPC. Our research group found that *circSETD3* acts as a molecular sponge to absorb *miR-615-5p* and *miR-1538*, which promotes the expression of MAREP1 and the metastasis of NPC [20]. *circARHGAP12* promotes the metastasis of NPC by regulating the Ezrin complex [17]. Furthermore, *circRNF13* inhibits the metastasis and proliferation of NPC via regulating the cell metabolic reprogramming [19]. However, research on the mechanism of circRNAs in the development of NPC is still insufficient.

In this study, we identified a large number of circRNAs through RNA sequencing of NPC clinical samples. We focused on *circCAMSAP1* because of its high expression in NPC and found it was significantly correlated with high TNM stages in NPC. To further explore the relationship between the expression of *circCAMSAP1* and the prognosis of NPC patients, we will conduct follow-up analyses of the cases, especially multicenter analyses with large samples. More samples should also be collected to verify whether *circCAMSAP1* can be used as a new marker for liquid biopsy of patients with nasopharyngeal cancer.

It has been reported that *circCAMSAP1* promotes tumor proliferation or metastasis in colorectal cancer [35], osteosarcoma [36], and liver cancer [37] through the competitive endogenous RNA (ceRNA) mechanism. In this study, we found that *circCAMSAP1* does not directly bind with the SERPINH1 or c-Myc proteins, but *circCAMSAP1* can directly bind to the *SERPINH1* 3’UTR, and *circCAMSAP1* stabilized *SERPINH1* expression by directly binding to its 3’UTR, ultimately promoting the expression of the SERPINH1 protein. Moreover, *circCAMSAP1* may be involved in various mechanisms in tumors.

SERPINH1, a collagen synthesis protein, is required for the correct folding and secretion of collagen. Although previous studies have shown that SERPINH1 is a potential molecular marker and therapeutic target for many tumors including gastric cancer [38], hepatocellular carcinoma [21], cervical squamous cell carcinoma [39], renal cell carcinoma [40], and head and neck squamous cell carcinoma [41], the mechanism by which SERPINH1 affects tumor progression through the production of collagen is unclear. In this study, we found that SERPINH1 could inhibit c-Myc ubiquitination-degradation. Furthermore, c-Myc, as a transcription factor, can regulate the expression of many proteins, play an important role in cell proliferation, cycle regulation, invasion, and metastasis, and affect the development of many tumors including NPC [25].

c-Myc directly promotes the expression of EMT signaling pathway-related proteins [42] and cell cycle-related proteins [43]. Meanwhile, c-Myc upregulates the expression of NPC-related proteins, such as BCAT1 [44], BMI1 [45] and BRD7 [46], to indirectly aggravate NPC development. The c-Myc-induced overexpression of non-coding RNAs contributes to the regulation of downstream genes, further enhancing the metastasis and proliferation of NPC [46–50]. c-Myc also increases the levels of metabolic-related proteins for the execution of metabolic reprogramming, which in turn leads to metastasis and proliferation [51, 52]. Furthermore, c-Myc interacts with signaling networks associated with NPC metastasis and proliferation, including the PI3K/Akt [53] and JNK/c-JUN [54, 55] pathways, exacerbating the malignant progression of NPC.

Since c-Myc has many target genes, we continued to analyze the differentially expressed proteins in NPC cells obtained from the proteomics platform after overexpression or knockdown of *circCAMSAP1*. Among them, *circCAMSAP1* up-regulated 97 proteins and down-regulated 146 proteins. The functional and signaling pathway enrichment were conducted through the Metascape database and DAVID database (Table S7). The results show that some proteins functionally enriched to cell-cell adherens junctions [56], the cell cycle, and G2/M checkpoints [57], whereas signaling pathway enrichment included the Rho GTPase signaling pathway [58], VEGFA/VEGFR signaling pathway [59], noncanonical NF-κB signaling [60], WNT/β-catenin signaling pathway [61], and PI3K/Akt signaling pathway [53], all of which are closely related to tumor metastasis and proliferation. In addition, the function of differential proteins is closely related to glycolysis/gluconeogenesis [19] and amino acid metabolism [62], promoting the malignant progression of tumors through metabolic reprogramming. Therefore, *circCAMSAP1* may either affect the expression of metastatic proliferation-related proteins and pathways through SERPINH1-c-Myc, or regulate metabolic reprogramming, which in turn affects the malignant progression of NPC. Further research concerning the specific mechanisms and targets of circCAMSAP1/SERPINH1/c-Myc axis should be addressed in the future.

In this study, we found that c-Myc promoted the transcription of *CAMSAP1* to form pre-mRNA and the expression of SRSF10. The high expression of SRSF10 could promote pre-mRNA splicing to form *circCAMSAP1*. The splicing process is usually completed by a complicated splicing complex [63]; we have identified that SRSF10 participates in the splicing of *circCAMSAP1*. Whether SRSF10 combines with other splicing factors or RBPs to form a splicing complex warrants further exploration.

## Conclusions

**Fig. 8:**
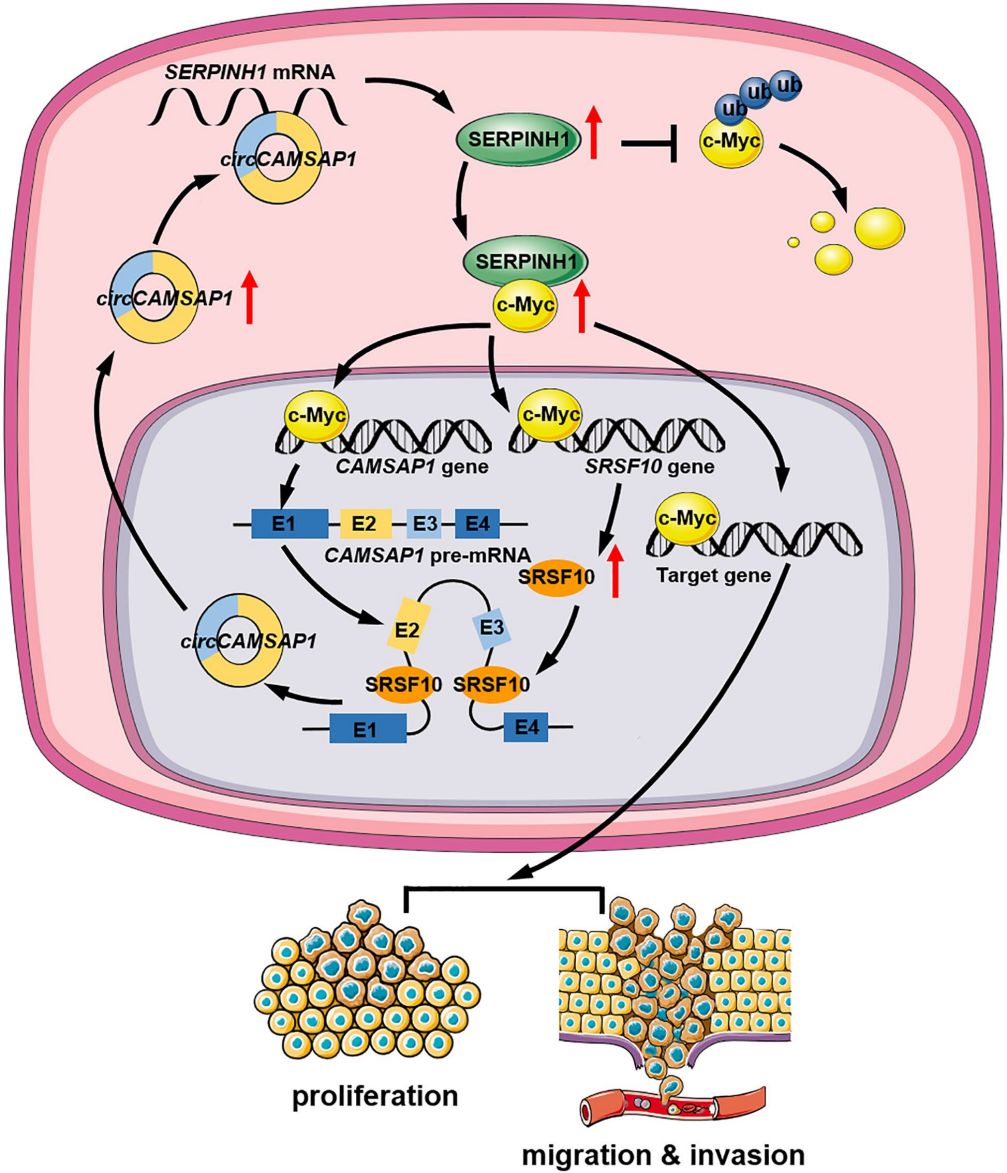
Schematic illustration of *circCAMSAP1* promoting proliferation and metastasis of NPC through a positive SERPINH1/c-Myc feedback. *circCAMSAP1* stabilizes *SERPINH1* expression by binding to the *SERPINH1* 3’UTR. The binding of SERPINH1 to c-Myc inhibits the ubiquitination and degradation of c-Myc, leading to *CAMSAP1* pre-mRNA transcription and back-splicing under the action of SRSF10, forming a positive feedback on *circCAMSAP1* production, resulting in proliferation, migration, and invasion of NPC

We reported the identification of a new circRNA, *circCAMSAP1* that has potent oncogenic activities in NPC (Fig. 8). Further, we demonstrated that *circCAMSAP1* promotes the growth and aggressiveness of NPC cells by stabilizing *SERPINH1* expression through binding to its *SERPINH1* 3’UTR. Additionally, the binding of SERPINH1 to c-Myc inhibited the ubiquitination-degradation of c-Myc. Meanwhile, c-Myc along with SRSF10 could promote *CAMSAP1* pre-mRNA transcript and back-splicing, and form a positive feedback on *circCAMSAP1* production, resulting in proliferation and metastasis of NPC. This study expands the understanding of circRNA function in NPC pathogenesis and suggests a novel circRNA as a potential biomarker and therapeutic target for NPC.

## Supplementary Information

**Additional file 1: Figure S1.** *circCAMSAP1* is highly expressed in NPC. A. Scatter plot of expression of *circCAMSAP1* in GSE68799 database. NPE, *n* = 4; NPC, *n* = 41. *p* = 0.2320. B. The RT-qPCR results showed that *circCAMSAP1* was related to the clinical stage and TN stage of patients with NPC in 12 NPE and 29 NPC samples. *, *p* < 0.05; **, *p* < 0.01; ****, *p* < 0.0001. C. The ISH results showed that *circCAMSAP1* is related to the clinical stage and TNM stage of patients with NPC in 29 NPE and 82 NPC samples. **, *p* < 0.01; ***, *p* < 0.001; ****, *p* < 0.0001. D. The expression of *circCAMSAP1* in NPC cell lines and immortalized NPE cell line NP69 detected by RT-qPCR. E. Sanger sequencing verified that *circCAMSAP1* is composed of reverse splicing of exons 2 and 3 of *CAMSAP1* mRNA, 425 bp, followed by RT-qPCR.

**Additional file 2: Figure S2.** *circCAMSAP1* promotes proliferation, migration, and invasion of NPC cells in vitro*.* A. The efficiency of overexpression or knockdown of *circCAMSAP1* in NPC cell lines detected by RT-qPCR. *, *p* < 0.05; **, *p* < 0.01; ***, *p* < 0.001; ****, *p* < 0.0001. B-D. Wound-healing assays (B), MTT assays (C), and the original data of flow cytometry analysis (D) in NPC cells after overexpression or knockdown of *circCAMSAP1*. Data are shown as means ± SD. *, *p* < 0.05; **, *p* < 0.01; ***, *p* < 0.001; ****, *p* < 0.0001.

**Additional file 3: Figure S3.** LC-MS/MS identification of downstream regulatory proteins by *circCAMSAP1.* A. Mass spectrometry data showed *circCAMSAP1* potential regulatory proteins in CNE2 cells after overexpression or knockdown of *circCAMSAP1*. B. The expression of *SERPINH1* in NPC microarrays GSE12452 and GSE53819. C. The expression of *SERPINH1* was measured in 29 NPC tissues and 12 non-cancerous NPE tissue samples by RT-qPCR. *β-actin* was used as an internal reference. *, *p* < 0.05. D. Correlation analysis of *circCAMSAP1* and *SERPINH1* mRNA expression in 29 NPC tissues. *p* < 0.0001. E. No binding between *circCAMSAP1* and SERPINH1 protein confirmed by RNA pull-down. F. *circCAMSAP1* was predicted to bind to and the *SERPINH1 *mRNA 3’UTR using the RNA22 software.

**Additional file 4: Figure S4.** SERPINH1 promotes proliferation, migration, and invasion of NPC cells in vitro*.* A-B. The efficiency of SERPINH1 overexpression or knockdown in NPC cell lines examined by RT-qPCR and western blotting. *, *p* < 0.05; **, *p* < 0.01; ***, *p* < 0.001; ****, *p* < 0.0001. C-E. Wound-healing assay (C), transwell invasion (D), and MTT assays (E) in NPC cells after overexpression or knockdown of SERPINH1. Data are shown as means ± SD. *, *p* < 0.05; **, *p* < 0.01; ***, *p* < 0.001; ****, *p* < 0.0001.

**Additional file 5: Figure S5.** *circCAMSAP1* promotes proliferation, migration, and invasion of NPC cells through SERPINH1. Wound-healing assay (A) and MTT assays (B) in NPC cells after knockdown or overexpression of *circCAMSAP1* and SERPINH1. Data are shown as means ± SD. *, *p* < 0.05; **, *p* < 0.01; ***, *p* < 0.001; ****, *p* < 0.0001.

**Additional file 6: Figure S6.** *circCAMSAP1* promotes c-Myc expression through SERPINH1 in NPC cells. A. The expression of c-Myc after overexpression or knockdown of SERPINH1 in NPC cell lines examined by western blotting. B. The expression of c-Myc in NPC cells after overexpression or knockdown of *circCAMSAP1* and SERPINH1 examined by western blotting. C. The expression of *c-Myc* mRNA in NPC cells after overexpression or knockdown of *circCAMSAP1* and SERPINH1 examined by RT-qPCR.

**Additional file 7: Figure S7.** *circCAMSAP1* inhibits c-Myc ubiquitination degradation through SERPINH1 in NPC cells. A. Western blotting analysis of the stability of c-Myc protein in SERPINH1 overexpressed NPC cell lines after treatment with CHX. B. Western blotting analysis of the ubiquitination of c-Myc protein after knockdown of SERPINH1 in NPC cell lines. C. The phosphorylation level of c-Myc T58 and S62 phosphorylation sites was measured by western blotting in NPC cell lines after overexpression or knockdown of SERPINH1. D. The phosphorylation level of c-Myc T58 and S62 phosphorylation sites was measured by western blotting in NPC cell lines after overexpression or knockdown of *circCAMSAP1* and SERPINH1.

**Additional file 8: Figure S8.** *circCAMSAP1* promotes c-Myc to play the role of transcription factor through SERPINH1 in NPC cells. A. The nuclear expression of c-Myc was examined in NPC cell lines after overexpression of SERPINH1 in NPC cell lines by western blotting. B. The transcript activity of c-Myc was measured in NPC cells after overexpression or knockdown of SERPINH1. ***, *p* < 0.001; ****, *p* < 0.0001.

**Additional file 9: Figure S9.** *circCAMSAP1* promotes the metastasis and proliferation of NPC through SERPINH1/c-Myc in vivo*.* A. H&E staining, ISH and immunohistochemistry detect the overexpression and knockdown efficiency of *circAMSAP1* and the expression of SERPINH1 and c-Myc in lung metastasis model in nude mice (left) and nude mouse subcutaneous tumor model (right). Scale bar = 20 μm. B-C. The expression of *circCAMSAP1*, SERPINH1, and c-Myc and the correlation analysis in nude mouse lung metastasis model and subcutaneous tumor model. *, *p* < 0.05; **, *p* < 0.01; ****, *p* < 0.0001.

**Additional file 10: Figure S10.** c-Myc combined with SRSF10 to form a positive feedback to promote the expression of *circCAMSAP1.* A. The efficiency of *c-Myc* overexpression or knockdown in NPC cell lines detected by RT-qPCR. *, *p* < 0.05; **, *p* < 0.01; ***, *p* < 0.001; ****, *p* < 0.0001. B. The efficiency of c-Myc overexpression or knockdown in NPC cell lines detected by western blotting. C. The expression of *CAMSAP1* mRNA was examined by RT-qPCR in NPC cells after overexpression or knockdown of c-Myc. *, *p* < 0.05; **, *p* < 0.01; ***, *p* < 0.001. D. The expression of *CAMSAP1* mRNA was analyzed in NPC microarrays GSE12452 and GSE53819. E. Bioinformatics analysis of the potential binding sites of c-Myc in the promoter region of *CAMSAP1*. F. The expression of *SRSF10* was analyzed in NPC microarrays GSE12452 and GSE53819. G. The efficiency of overexpression or knockdown of *SRSF10* in NPC cell lines by RT-qPCR. **, *p* < 0.01; ***, *p* < 0.001; ****, *p* < 0.0001.

**Additional file 11: Table S1.** Clinicopathological data for 29 NPC tissues and 12 non-neoplastic NPE tissue samples used for RT-qPCR.

**Additional file 12: Table S2.** Clinicopathological data of 82 paraffin-embedded NPC tissues and 29 non-neoplastic NPE tissues for in situ hybridization.

**Additional file 13: Table S3:** Probes for siRNAs, ISH, FISH, RNA pull-down and circRIP, and primers for circRIP, RIP, ChIP experiment, the overexpression vectors, the luciferase reporter vectors, and RT-qPCR.

**Additional file 14: Table S4.** List of antibodies for immunohistochemistry, western blotting, immunofluorescence, RNA pull-down, Co-immunoprecipitation, or ChIP analyses.

**Additional file 15: Table S5.** The top 20 circRNAs according to their RPM values in 41 NPC tissues from the GSE68799 database.

**Additional file 16: Table S6.** Mass spectrometry identification of proteins positively regulated by *circCAMSAP1*.

**Additional file 17: Table S7.** Functional and signaling pathway enrichment of the differentially expressed proteins possibly regulated by *circCAMSAP1*.

## Data Availability

All data that support the findings of this study are available from the corresponding authors upon reasonable request.

## Abbreviations

FISH: Fluorescence in situ hybridization
GEO: Gene Expression Omnibus
ISH: In situ hybridization
NPC: Nasopharyngeal carcinoma
NPE: Nasopharyngeal epithelial
RPM: Reads per million
WT: Wild type
ChIP: chromatin immunoprecipitation
CHX: cycloheximide
CircRNA: Circular RNA
DAPI: 6-diamidino-2-phenylindole
EV: empty vector
GAPDH: Glyceraldehyde-3-phosphate dehydrogenase
MTT: 3-(4,5-dimethylthiazol-2-yl)-2,5-diphenyl-2H-tetrazolium bromide
NC: scrambled negative control
OE: overexpression vector
RBP: RNA-binding proteins
RIP: RNA immunoprecipitation
TFA: trifluoroacetic acid
TNM: tumor (T), nodes (N), and metastases (M)
